# Polymorphism rs3733591 of the *SLC2A9* gene and metabolic syndrome affect gout risk in Taiwan Biobank subjects

**DOI:** 10.3389/fgene.2024.1374405

**Published:** 2024-04-16

**Authors:** Chun-Nan Lin, Chien-Chang Ho, Pao-Chun Hsieh, Chih-Hsuan Hsiao, Oswald Ndi Nfor, Yung-Po Liaw

**Affiliations:** ^1^ Department of Emergency Medicine, Chung-Kang Branch, Cheng Ching Hospital, Taichung, Taiwan; ^2^ Department of Physical Education, Fu Jen Catholic University, New Taipei, Taiwan; ^3^ Research and Development Center for Physical Education, Health, and Information Technology, Fu Jen Catholic University, New Taipei, Taiwan; ^4^ Department of Obstetrics and Gynecology, Chung-Kang Branch, Cheng Ching Hospital, Taichung, Taiwan; ^5^ Department of Public Health and Institute of Public Health, Chung Shan Medical University, Taichung, Taiwan; ^6^ Department of Medical Imaging, Chung Shan Medical University Hospital, Taichung, Taiwan

**Keywords:** metabolic syndrome, gout, polymorphism, genetics, osteoarthritis

## Abstract

**Background:**

Over the past few decades, gout and diseases like metabolic syndrome (MetS) have become more prevalent. Attempts have been made in Taiwan to identify the genes responsible for gout. A few gene loci, among them *SLC2A9*, have been identified using Taiwan Biobank (TWB) data. We, therefore, examined whether MetS could also account for the association between polymorphism *SLC2A9* rs3733591 and gout.

**Methods:**

The final analysis consisted of 73,558 subjects, of whom 2,709 had gout. To estimate the likelihood of gout occurrence based on rs3733591 and MetS, we used logistic regression models.

**Results:**

Rs3733591-TC + CC compared to TT genotype was associated with gout (OR, 1.15; 95% CI, 1.06–1.25). Also associated with gout was MetS (OR, 1.21; 95% CI, 1.10–1.33). A significant interaction was seen between rs3733591 and MetS (*p*-value = 0.039). Using rs3733591-TT/no MetS as the reference group, the ORs (95% CI) for gout was 1.24 (1.11–1.38) for TC + CC/no MetS, 1.35 (1.17–1.56) for TT/MetS, and 1.39 (1.22–1.58) for TC + CC/MetS. However, subgroup analysis defined by sex showed no significant associations in women.

**Conclusion:**

In summary, metabolic syndrome and *SLC2A9* rs3733591 genotypes were interactively associated with gout in Taiwanese men, but not women.

## Introduction

Gout is the most common form of inflammatory arthritis with the worst affected groups being elderly people, men, and racial and ethnic minorities (Singh and Gaffo, 2020). Recent years have seen an increase in the prevalence of gout and other diseases such as MetS and obesity (Pascart and Lioté, 2019). Taiwan is one of the countries where men are more likely to suffer from gout (Singh and Gaffo, 2020). About 1%–6.8% of people across the globe (Dehlin et al., 2020) and 3.8% in Taiwan (5.2% in men and 2.3% in women) are affected by gout (Kuo et al., 2013; Cox et al., 2021). It is mostly caused by hyperuricemia (elevated blood urate concentrations). Urate-lowering medications have been frequently used to treat the disease, though further efforts are needed to improve adherence to these medications (Pascart and Lioté, 2019; Dehlin et al., 2020). Previous epidemiological studies have also linked gout with MetS among Taiwanese (Wei et al., 2015) and US adults (Yoo et al., 2011). Furthermore, studies in other populations (RHO et al., 2004; Choi et al., 2007; Yoo et al., 2011; Jung et al., 2018) indicated that patients with gout had a greater prevalence of MetS than the general population.

As noted above, gout is linked to serum urate, whose heritability is estimated at 40%–70% as previously described (Wilk et al., 2000; Yang et al., 2005; Nath et al., 2007; Narang et al., 2019)*.* According to our knowledge, several genes have been studied in relation to hyperurecemia and gout. Among them is the *SLC2A9* gene, which is one of the key urate transporters in the kidney and gut that regulate serum urate. It is found on chromosome 4p16 and encodes the *glucose transporter 9* (*GLUT9*), which is crucial for urate reuptake (Stiburkova et al., 2012; Pavelcova et al., 2020). Compared to men, variation in *SLC2A9* is believed to have the greatest effect on serum urate in women (i.e., approximately 6% as opposed to 2% in men) (Riches, 2012). Topless et al. observed that the *SLC2A9* variant affected serum urate more in premenopausal women than in postmenopausal women (Topless et al., 2015). So far, the *SLC2A9* gene has shown the strongest association with uric acid levels among Asians but not much has been done to investigate it in Taiwan. Moreover, replication studies have shown conflicting results on *SLC2A9* polymorphisms in gout and serum urate (Lukkunaprasit et al., 2020).

*SLC2A9* rs3733591 is among polymorphisms found to be common in people with hyperurecemia and gout (Tu et al., 2010; Zhang et al., 2016; WT et al., 2018; Pavelcova et al., 2020) even though contrary results have also been published. For instance, an earlier study on the Minnan population did not find an association between the variant and gout risk (Zheng et al., 2016). In another study, Hollis-Moffatt et al. were not able to replicate associations of rs3733591 with gout in Caucasians and Western/Eastern Polynesians (Hollis-Moffatt et al., 2011). In the authors’ view, the variant has a weaker effect on gout in these populations than it does in Asian populations. According to Hung et al., the variant did not appear to increase gout susceptibility in Vietnamese populations (Hung et al., 2022).

There is evidence suggesting a link between gout and metabolic syndrome (González-Senac et al., 2014; Eun et al., 2022; Eun et al., 2023). Both conditions share common risk factors, such as obesity, insulin resistance, hypertension, and dyslipidemia. Exploring candidate genes and their variants linked to serum urate levels aids in regulating the metabolic processes involved in both urate production and removal (Köttgen et al., 2013): This knowledge could potentially inform strategies for treating and preventing gout.

The genetic basis of gout in the Taiwanese population has been a subject of investigation, with efforts focused on identifying relevant genetic loci. However, research thus far, particularly utilizing data TWB, has only yielded a limited number of identified loci, notably including *SLC2A9*. Despite these efforts, large-scale replication studies confirming genome-wide significant associations in this population remain scarce (Ko, 2022). A case-control study (Tu et al., 2018), albeit with limited sample size, aimed to determine the positive predictive value for gout in Taiwan. This study recruited individuals from both Han Chinese and indigenous populations across various communities through hospital settings. Notably, the study found that rs3733591 was not associated with gout in the indigenous population, in contrast to its association in the Han population. Surprisingly, despite its potential relevance, research focusing on this SNP in the context of gout in Taiwan has been minimal. Given the gap in understanding the role of rs3733591 in gout within the Taiwanese population, we sought to address this by leveraging the extensive data available from the TWB. Thus, we examined the association between polymorphism rs3733591 and gout, while also assessing whether this association could be attributed to MetS.

## Materials and methods

### Study design and participants

This study utilized data from the TWB resource, where demographic, lifestyle, biochemistry, and genotype data were collected through biobank questionnaires. At the beginning of the study, data from 76,229 participants were available. Exclusion criteria included subjects with incomplete information (*n* = 2,671). Ultimately, 73,558 subjects were analyzed, 2,709 of whom had gout. Informed written consent was obtained from all TWB participants at the time of recruitment. The institutional Review Board of Cheng-Ching General Hospital approved this study (HP210007). All methods were performed in accordance with the relevant guidelines and regulations.

### Genotyping and quality control

Whole-genome genotyping was performed on the Biobank participants using the Axiom Genome-Wide Array Plate chip system (Affymetrix Inc., Santa Clara, CA, United States). This study examined *SLC2A9* rs3733591 of the urate transporter gene. During quality control, we ensured that the variant rs3733591 had a call rate of >95 percent, a *p*-value of >0.00001 for the Hardy-Weinberg equilibrium test, and a minor allele frequency of >0.01.

### Disease definition and covariates

The outcome of our interest was self-reported gout, with exposures including rs3733591 and MetS, which was defined according to the revised National Cholesterol Education Program (NCEP) Adult Treatment Panel III (ATP III) criteria as proposed by the Health Promotion Administration in Taiwan (Health Promotion Administration, 2007; Chen et al., 2020). MetS was defined as having at least three of the following conditions: (1) hypertension (systolic blood pressure ≥130 mmHg or diastolic blood pressure ≥85 mmHg); (2) abdominal obesity (waist circumference: men ≥90cm, women ≥80 cm); (3) low high-density lipoprotein cholesterol (men <40 mg/dL, women <50 mg/dL); (4) hyperglycemia (fasting blood glucose ≥100 mg/dL) or (5) triglyceride ≥150 mg/dL. Hyperlipidemia was defined based on a self-report questionnaire or triglyceride and total cholesterol levels of over 150 mg/dL and 200 mg/dL, respectively. The adjusted variables included sex, body mass index (BMI, determined as weight divided by height squared (kg/m^2^), smoking, drinking, exercise, hypertension, and hyperuricemia. Those who have smoked continuously for at least 6 months are classified as current smokers and those who have quit smoking for at least 6 months as former smokers. Those who consumed more than 150 mL/week for 6 months were considered current drinkers, and those who stopped drinking for 6 months were considered former drinkers. Exercise was defined as engaging in physical activity for 30 min or more at least three times a week. Historically, hyperuricemia was defined as uric acid levels over 6 mg/dL in females and 7 mg/dL in males.

### Statistical analysis

Both continuous and categorical variables were tested using Student's t-test and Chi-square test. In order to estimate the likelihood of gout based on rs3733591 and MetS, we used logistic regression. There was also a test to determine whether rs3733591 interacts with MetS. The statistical software used in this study is SAS 9.4 (SAS Institute, Cary, NC, United States) and PLINK 1.90 beta.

## Results

Table 1 outlines the characteristics of subjects from TWB. Among the subjects, 70,849 were healthy and 2,709 had gout. Among subjects with gout, 259 (9.56%) were females and 2,450 (90.44%) were males. As shown in Table 2, factors significantly associated with gout were rs3733591-TC + CC compared to TT genotype (OR, 1.15; 95% CI, 1.06–1.25), MetS (OR, 1.21; 95% CI, 1.10–1.33), male compared to female sex (OR, 14.26 95% CI, 12.42–16.36), hyperuricemia (OR, 4.47.95% CI, 4.09–4.87), hyperlipidemia (OR, 2.51, 95% CI, 2.25–2.80), and hypertension (OR, 1.66, 95% CI, 1.50–1.83), respectively. The sex-related risk of gout among study subjects is also shown. Compared to the TT genotype, the OR (95% CI) for gout in TC + CC males was 1.16 (1.06–1.27) and 1.04 (0.81–1.34) in TC + CC females. Hyperuricemia, increasing age, hyperlipidemia, hypertension, and obesity were all associated with gout in both men and women. There was an interaction between variant rs3733591 and MetS (*p*-value = 0.039). Due to interaction, we further stratified our analyses using rs3733591-TT/no MetS as the reference group, as shown in Table 3. In the general population, the ORs (95% CI) were 1.24 (1.11–1.38) for TC + CC/no MetS, 1.35 (1.17–1.56) for TT/MetS, and 1.39 (1.22–1.58) for TC + CC/MetS. In a sex-based stratification, female subjects showed no significant associations, unlike their male counterparts (Table 3).

**TABLE 1:** Baseline characteristics of subjects with and without gout.

	No gout (*n* = 70,849)	Gout (*n* = 2,709)	*p*-value
SLC2A9 rs3733591 (n, %)			<0.001
TT	30,902 (43.62)	1,047 (38.65)	
TC + CC	39,947 (56.38)	1,662 (61.35)	
Metabolic syndrome (n, %)			<0.001
no	55,882 (78.87)	1,547 (57.11)	
yes	14,967 (21.13)	1,162 (42.89)	
Sex (n, %)			<0.001
female	48,081 (67.86)	259 (9.56)	
male	22,768 (32.14)	2,450 (90.44)	
Age	50.17 ± 10.68	53.06 ± 10.31	<0.001
Body mass index (kg/m^2^)			<0.001
Normal weight	37,002 (52.23)	612 (22.59)	
overweight	19,506 (27.53)	950 (35.07)	
obese	14,341 (20.24)	1,147 (42.34)	
Smoking (n, %)			<0.001
never	57,785 (81.56)	1,456 (53.75)	
former	6,964 (9.83)	736 (27.17)	
current	6,100 (8.61)	517 (19.08)	
Drinking (n, %)			<0.001
no	65,235 (92.08)	2084 (76.93)	
yes	5,614 (7.92)	625 (23.07)	
Exercise (n, %)			<0.001
no	42,097 (59.42)	1,445 (53.34)	
yes	28,752 (40.58)	1,264 (46.66)	
Hyperuricemia (female ≥6 mg/dL, male ≥7 mg/dL)			<0.001
no	56,894 (80.30)	967 (35.70)	
yes	13,955 (19.70)	1742 (64.30)	
Hyperlipidemia (n, %)			<0.001
no	65,822 (92.90)	2057 (75.93)	
yes	5,027 (7.10)	652 (24.07)	
Hypertension (n, %)			<0.001
no	62,521 (88.25)	1783 (65.82)	
yes	8,328 (11.75)	926 (34.18)	

Abbreviation: TT, TC, and CC: genotypes of rs3733591 polymorphism. Continuous variables displayed as mean ± standard deviation. Categorical variables displayed as number (percentage).

**TABLE 2:** Odds ratio for gout in study population stratified by sex.

	All subjects			Female (*n* = 48,340)			Male (*n* = 25,218)		
	OR	95%CI	*p*-value	OR	95%CI	*p*-value	OR	95%CI	*p*-value
rs3733591 (ref: TT)									
TC + CC	1.15	1.06–1.25	0.001	1.04	0.81–1.34	0.734	1.16	1.06–1.27	<0.001
Metabolic syndrome (ref: no)									
yes	1.21	1.10–1.33	<0.001	1.12	0.85–1.49	0.428	1.22	1.11–1.35	<0.001
Sex (ref: female)									
male	14.26	12.42–16.36	<0.001	-	-	-	-	-	-
Age	1.02	1.01–1.02	<0.001	1.03	1.02–1.05	<0.001	1.02	1.01–1.02	<0.001
BMI (ref: normal weight)									
overweight	1.24	1.11–1.38	<0.001	1.31	0.94–1.81	0.108	1.22	1.08–1.38	0.001
obese	1.56	1.38–1.75	<0.001	1.91	1.38–2.65	<0.001	1.51	1.33–1.71	<0.001
Smoking (ref: never)									
former	0.99	0.89–1.10	0.828	1.32	0.64–2.74	0.449	0.98	0.88–1.09	0.736
current	1.03	0.92–1.16	0.603	2.72	1.58–4.69	<0.001	1.00	0.88–1.12	0.955
Drinking (ref: no)									
yes	1.17	1.05–1.30	0.005	0.91	0.45–1.85	0.801	1.18	1.06–1.31	0.003
Exercise (ref: no)									
yes	1.08	0.99–1.18	0.100	1.09	0.84–1.42	0.510	1.07	0.98–1.18	0.137
Hyperuricemia (ref: no)									
yes	4.47	4.09–4.87	<0.001	4.24	3.24–5.55	<0.001	4.44	4.05–4.87	<0.001
Hyperlipidemia (ref: no)									
yes	2.51	2.25–2.80	<0.001	2.71	2.00–3.67	<0.001	2.47	2.20–2.78	<0.001
Hypertension (ref: no)									
yes	1.66	1.50–1.83	<0.001	1.62	1.20–2.19	0.001	1.66	1.49–1.85	<0.001
Rs3733591*MetS	*p = 0.039*			*p =* 0.956			*p =* 0.026		

Abbreviations: MetS, metabolic syndrome, TT, TC and CC, genotypes of rs3733591 polymorphism. OR, odds ratio. 95% CI, 95% confidence interval.

**TABLE 3:** Association of gout with rs3733591 genotypes and MetS stratified by sex.

	All participants			Female (*n* = 48,340)				Male (*n* = 25,218)			
	OR	95%CI	*p*-value	OR		95%CI	*p*-value	OR		95%CI	*p*-value
rs3733591 & MetS (ref: TT & no MetS)											
TC + CC & no MetS	1.24	1.11–1.38	<0.001	1.04		0.75–1.44	0.823	1.27		1.13–1.42	<0.001
TT & MetS	1.35	1.17–1.56	<0.001	1.11		0.73–1.69	0.620	1.39		1.20–1.61	<0.001
TC + CC & MetS	1.39	1.22–1.58	<0.001	1.17		0.80–1.70	0.410	1.43		1.24–1.63	<0.001
Sex (ref: female)											
male	14.25	12.41–16.36	<0.001	-		-	-	-		-	-
Age	1.02	1.01–1.02	<0.001	1.03		1.02–1.05	<0.001	1.02		1.01–1.02	<0.001

Abbreviations: MetS, metabolic syndrome, TT, TC and CC, genotypes of rs3733591 polymorphism. OR, odds ratio. 95% CI, 95% confidence interval. Adjusted for BMI, smoking, alcohol consumption, exercise, hypertension, and hyperuricemia.

## Discussion

Gout, a prevalent ailment among native Taiwanese, has been linked to numerous loci associated with urate concentrations. In our investigation, we sought to ascertain the impact of MetS and the presence of *SLC2A9*, a gene strongly linked to urate concentrations, on gout development in Taiwan. Our findings revealed that in the general model, the presence of SLC2A9 rs3733591-TC + CC genotypes, compared to rs3733591-TT, along with MetS, exhibited independent associations with gout. Notably, gender disparities emerged regarding MetS and the rs3733591-TC + CC genotype, with significant odds ratios observed solely among men.

The C-allele of SNP rs3733591 has been identified as the risk allele for gout among Han Chinese and Solomon Islanders (Tu et al., 2010), underscoring its significance as a genetic determinant of uric acid concentrations and gout in Taiwan. According to Tu et al., this variant is an important genetic checkpoint for uric acid concentrations and gout in Taiwan. As seen earlier, rs3733591 is believed to have a weaker effect in Caucasians and Polynesians than it does in Asians (Hollis-Moffatt et al., 2011). Despite these, our logistic regression model revealed a significant interaction between the variant and MetS. However, in stratified analyses, this interaction demonstrated a more pronounced association with gout in men than in women, warranting further elucidation of underlying mechanisms.

The genetic variant, rs3733591 resides within the *SLC2A9* gene. A comprehensive examination spanning the entire *SLC2A9* locus would have been imperative to ascertain whether rs3733591 ranks among the primary SNPs associated with gout and serum urate levels at this locus. The complexity of the *SLC2A9* locus, potentially harboring multiple distinct signals of association attributable to various causal variants and compounded by population structure, underscores the need for thorough investigation.

While acknowledging the intricacies of the *SLC2A9* locus and the possibility of multiple independent signals of association, our study sought to validate and expand upon previous findings. However, to evaluate the significance of rs3733591 as a principal variant within the *SLC2A9* gene linked to gout and serum urate levels, we employed AI (artificial intelligence) models (data not presented) that encompassed seven *SLC2A9* loci (rs3733591, rs16890979, rs1014290, rs6449213, rs6855911, rs12498742, rs3775948) previously associated with these conditions. Our analysis demonstrated that rs3733591 ranked among the top three SNPs in terms of relative importance within the Gradient Boosting champion model. This helps alleviate concerns regarding potential artefacts arising from complex linkage disequilibrium (LD) patterns at this locus.

Both our general and sex-stratified models indicated that hyperuricemia was associated with a four-fold increased risk of gout. This aligns with previous findings highlighting the requisite presence of hyperuricemia and distinct inflammatory mechanisms for gout development (Chen et al., 2018). Acute inflammation in gout patients ensues from the accumulation of monosodium urate (MSU) crystals, which then interact with macrophages to trigger activation of the NOD-, LRR- and pyrin domain-containing protein 3 (NLRP3) inflammasome and release of Interleukin-1 β (IL-1β), which is linked to gout flare (Chen et al., 2018).

Furthermore, our analysis revealed no gender disparities in certain gout risk factors such as obesity, hypertension, and advancing age. Notably, alcohol consumption demonstrated a significant association with gout solely among men, despite previous indications (Wang et al., 2013) suggesting a dose-dependent effect of alcohol consumption on gout development.

In the present investigation, the absence of data regarding medication utilization represents a notable limitation. Furthermore, the unavailability of dietary information, including purine intake, within our chosen data source complicates the assessment of potential dietary influences on our findings. Additionally, the duration of illness was not ascertainable from the questionnaires administered within the Biobank database. Moreover, our identification of gout relied solely on self-reported data, which introduces the possibility of misclassification and may not fully capture the prevalence of gout within the broader population. Lastly, the inherent limitations of a cross-sectional study design impede the establishment of causal relationships among the variables under examination.

## Conclusion

In conclusion, we found that MetS and rs3733591 genotypes exhibited a stronger interactive association with gout in Taiwanese men but not in women. Understanding the discovery, potential causality, and linkage disequilibrium (LD) patterns of rs3733591 and other variants within the *SLC2A9* gene is crucial for elucidating their roles in gout susceptibility and metabolic syndrome, as well as their interactions with environmental factors. Further research, including functional studies and replication in diverse populations, is needed to validate and characterize these genetic associations comprehensively.

## Data Availability

The data analyzed in this study is subject to the following licenses/restrictions: The data that support the findings of this study are available from Taiwan Biobank but restrictions apply to the availability of these data, which were used under license for the current study, and so are not publicly available. Data are however available from the corresponding authors upon reasonable request and with permission of Taiwan Biobank. Requests to access these datasets should be directed to liawyp@csmu.edu.tw.
